# Mitogen- and stress-activated Kinase 1 mediates Epstein-Barr virus latent membrane protein 1-promoted cell transformation in nasopharyngeal carcinoma through its induction of *Fra-1* and *c-Jun* genes

**DOI:** 10.1186/s12885-015-1398-3

**Published:** 2015-05-10

**Authors:** Binbin Li, Zheng Wan, Guoliang Huang, Zunnan Huang, Xiangning Zhang, Dan Liao, Shengqun Luo, Zhiwei He

**Affiliations:** 1 Department of Pathophysiology, Guangdong Medical College, Dongguan, Guangdong, 523808 China; 2 Key Laboratory for Medical Diagnostics of Guangdong Province, Sino-American Cancer Research Institute, Guangdong Medical College, Dongguan, Guangdong 523808 China

**Keywords:** MSK1, NPC, EBV-LMP1, Transformation, Fra-1, c-Jun

## Abstract

**Background:**

Mitogen- and Stress-Activated Kinase 1 (MSK1) is a nuclear kinase that serves as active link between extracellular signals and the primary response of gene expression. However, the involvement of MSK1 in malignant transformation and cancer development is not well understood. In this study, we aimed to explore the role of MSK1 in Epstein-Barr virus (EBV) latent membrane protein 1 (LMP1)-promoted carcinogenesis of nasopharyngeal carcinoma (NPC).

**Methods:**

The level of MSK1 phosphorylation at Thr581 was detected by the immunohistochemical analysis in NPC tissues and normal nasopharynx tissues, and its correlation with LMP1 was analyzed in NPC tissues and cell lines. Using MSK1 inhibitor H89 or small interfering RNA (siRNA)-MSK1, the effects of MSK1 on LMP1-promoted CNE1 cell proliferation and transformation were evaluated by CCK-8 assay, flow cytometry and focus-forming assay respectively. Furthermore, the regulatory role of MSK1-mediated histone H3 phosphorylation at Ser10 on the promoter activity and expression of *Fra-1* or *c-Jun* was determined by reporter gene assay and western blotting analysis.

**Results:**

Immunohistochemical analysis revealed that the level of MSK1 phosphorylation at Thr581 was significantly higher in the poorly differentiated NPC tissues than that in normal nasopharynx tissues (*P* < 0.001). Moreover, high level of phosphorylated MSK1 was positively correlated with the expression of LMP1 in NPC tissues (r = 0.393, *P* = 0.002) and cell lines. MSK1 inhibitor H89 or knockdown of MSK1 by siRNA dramatically suppressed LMP1-promoted CNE1 cell proliferation, which was associated with the induction of cell cycle arrest at G_0_/G_1_ phase. In addition, the anchorage-independent growth promoted by LMP1 was blocked in MSK1 knockdown cells. When the activity or expression of MSK1 was inhibited, LMP1-induced promoter activities of *Fra-1* and *c-Jun* as well as their protein levels were greatly reduced. It was found that only H3 WT, but not mutant H3 S10A, dramatically increased LMP1 induction of *Fra-1* and *c-Jun* genes compared with mock cells.

**Conclusion:**

Increased MSK1 activity is critically important for LMP1-promoted cell proliferation and transformation in NPC, which may be correlated with its induction of *Fra-1* and *c-Jun* through phosphorylation of histone H3 at Ser10.

## Background

Nasopharyngeal carcinoma (NPC) is a particularly common tumor in areas of southern China and South-East Asia, reaching a peak incidence of 20-30 cases per 100,1000 per year [1]. Its occurrence involves the interaction of host genetic alterations with environmental factors, especially infection by Epstein-Barr virus (EBV) [2, 3]. EBV-encode latent membrane protein 1 (LMP1) is essential for the maintenance of latent infection and EBV-mediated malignant transformation [3, 4]. The C-terminal tail of LMP1 provides docking sites for the recruitment and activation of signaling adapter proteins, which triggers various downstream oncogenic signaling pathways, such as NF-κB, MAPK, PI3K and JAK/STAT pathways [5]. However, it is largely unknown what key effector proteins are induced and essentially promote cell transformation in the LMP1-triggered signaling events.

Mitogen- and stress-activated kinase 1 (MSK1) is a nuclear serine/threonine protein kinase that is mainly activated by both ERKs and p38 MAPKs. Because of its dual activation mode, MSK1 is able to integrate various physiological and pathological stimuli including mitogenic signals, cellular stress and pro-inflammatory cytokines [6]. MSK1 has been recognized as a versatile kinase regulating gene transcription at multiple levels. MSK1 directly phosphorylates various transcription factors, including cAMP-responsive element binding protein (CREB), activating transcription factor 1 (ATF1), signal transducers and activators of transcription 3 (Stat3), the p65 subunit of NF-_κ_B, thereby alters their ability of binding to target DNA or recruitment of required coactivator [7–9]. MSK1 also mediates the phosphorylation of chromatin protein histone H3 and high mobility group 14 (HMG14), which induces chromatin relaxation and contributes to gene activation, termed as “nucleosomal response” [10]. MSK1-mediated histone H3 phosphorylation at Ser10 is critical for epidermal growth factor (EGF), 12-O-tetradecanoylphorbol-13-acetate (TPA) and oncogene (e.g. H-*ras*)-induced cell transformation, which is associated with the induction of immediate-early (IE) genes such as *c-Fos*, *c-Jun* and *c-Myc* [11–13]. Overactive Ras-MAPK pathway and elevated MSK1 activity were observed in various cancerous tissues and cell lines [14, 15]. MSK1 is responsible for histone H3 phosphorylation of estrogen-responsive *Trefoil Factor-1* (*TFF-1*) promoter in breast cancer MCF-7 cells [16]. MSK1 also regulates transcriptional activation of NF-_κ_B-dependent pro-inflammatory genes in response to cigarette smoke [17]. These studies suggested that MSK1 might play an important role in carcinogenesis through aberrant histone modifications and transcriptional regulation. Our previous study demonstrated that phosphorylation of histone H3 at Ser10 mediated by MSK1 might be a crucial epigenetic change in LMP1-promoted carcinogenesis of NPC [18]. However, it is not completely clear whether the activation of MSK1 directly affects LMP1-promoted cell proliferation and transformation in NPC.

In the present study, we found that MSK1 was abnormally activated in both primary NPC tissues and NPC cell lines, and closely related to the expression of LMP1. Moreover, MSK1 activity was required for LMP1-promoted cell proliferation and transformation of NPC, which was associated with the induction of *Fra-1* and *c-Jun* by phosphorylation of histone H3 at Ser10. These findings provide a better understanding to the importance of MSK1-mediated nucleosomal response in the LMP1-induced malignant transformation and carcinogenesis.

## Methods

### Patients, tissue specimens and cell lines

Nasopharyngeal carcinoma tissue microarray (catalog no. NPC961) was from US Biomax (Rockville, MD), including 33 cases of poorly differentiated NPC tissues, 26 cases of adjacent normal tissues, and 10 cases of normal nasopharyngeal tissues. In addition, 20 cases of poorly differentiated NPC tissues were obtained from the First Affiliated Hospital of Guangdong Medical College, Zhanjiang, China. The patients received no other therapies, such as radiation or chemotherapy, prior to operation. All samples were confirmed by pathological examination and staging was performed according to the 1997 NPC staging system of the UICC. In the 53 NPC cases, there were 40 male and 13 female with age ranging from 26 to 62 years (median, 43.9 years). Informed consent was obtained from all patients, and this study was approved by the Institutional Ethics Committee of Guangdong Medical College.

CNE1 cells, an EBV-negative and well-differentiated human NPC cell line, were cultured in RPMI 1640 medium supplemented with 10 % fetal bovine serum (GIBCO, Carlsbad, CA, USA). CNE1G (CNE1 stably transfected with PAT-GFP) and CNE1GL (CNE1 stably transfected with PAT-GFP-LMP1) cells were provided by Dr. Xiaoyi Chen, Guangdong Medical College [19], and were maintained in completed RPMI 1640 medium described above, containing 0.5 μg/ml puromycin (Sigma-Aldrich, St. Louis, MO, USA).

### Plasmids, transfection and establishing stable cell lines

To construct the siRNA-mock (si-mock) or siRNA-MSK1 (si-MSK1), the mU6pro vector (a gift from Dr. Zigang Dong, Hormel Institute, University of Minnesota, Austin, Minnesota, USA) was digested with XbaI and BbsI. The annealed synthetic primers (si-mock: 5′-TTTGACTACCGTTGTTATAGGTGTTCAAGAGACACCTATAACAACGGTAGTTTTTT-3′ and antisense 5′- CTAGAAAAAAACTACCGTTGTTATAGGTGTCTCTTGAACACCT ATAACAACGGTAGT; si-MSK1: sense 5′-TTTGAGACCTAATTCAGCGTCTTTTCAAG AGAAAGACGCTGAATTAGGTCTTTTTT-3′ and antisense 5′-CTAGAAAAAAGACCT AATTCAGCGTCTTTCTCTTGAAAAGACGCTGAATTAGGTCT-3′) were then introduced following the recommending protocols. The recombinant plasmids were confirmed by agarose gel electrophoresis and DNA sequencing. The plasmids were transfected into CNE1 cells using JetPEI (Polyplus, llkirch) according to the manufacturer’s protocol. Stable CNE1 cells expressing si-mock or si-MSK1 were established with pcDNA6.0/myc-HisB as selection marker. Transfected cells were selected in medium containing 2 μg/ml blasticidin (Sigma-Aldrich, St. Louis, MO), and the expression level of MSK1 was confirmed by Western blotting analysis.

The pcDNA3.0 and pcDNA3.0-LMP1 vectors were kindly provide by Dr Ellen Cahir- McFarland, Brigham and Women’s Hospital, Boston, Massachusetts, USA. AP-1 reporter vector pRTU14 was kindly provided by Dr ArndKieser, Helmholtz ZentrumMünchen, Munich, Germany [20]. To construct the *Fra-1* and *c-Jun* promoter luciferase reporter vectors, DNA fragments of 5′-flanking region of the human *Fra-1* gene (-379 to -238) [21] and *c-Jun* gene (-117 to -50) [22] were synthesized and inserted into a basal promoter luciferase reporter vector (pGL3) respectively. The pcDNA6.0/myc-His B-histone H3 wide-type (pcDNA6.0-H3 WT) and pcDNA6.0/myc-His B-histone H3 S10A mutant (pcDNA6.0-H3 S10A) were constructed as reported previously [18]. pcDNA6.0-H3 WT or pcDNA6.0-H3 S10A stably transfected CNE1 cells were selected in medium containing 2 μg/ml blasticidin. Expression of vectors was confirmed with an antibody against the His epitope by Western blotting analysis.

### Immunohistochemical staining

Fixed tissue samples were sectioned (4 μm), deparaffinized, rehydrated, and subjected to heat-induced antigen retrieval in sodium citrate buffer (0.01 M, pH 6.0). Endogenous peroxidase activity and non-specific antigen were blocked with 3 % hydrogen peroxide and normal goat serum. The sections were incubated with the primary antibodies against phosphorylated MSK1 (Thr581) or LMP1 overnight at 4 °C. HRP-conjugated secondary antibodies (ChemMate Envision Detection Kit, DAKO) were applied onto the sections and incubated for 30 min at room temperature. 10 % normal goat serum was used to replace primary antibodies as a negative control.

The slides were reviewed and scored independently by two pathologists. Phosphorylated MSK1 at Thr581 was expressed in the cell nucleus and the staining was scored according to its intensity (0, no staining; 1, weakly staining; 2, moderately staining; 3, strongly staining) and the percentage of positive cells (0, <5 % positive cells; 1, 5 %-25 % positive cells; 2, 26-50 % positive cells; 3, >50 % positive cells). The final expression score was calculated from “intensity score” multiplied by “percentage”. For statistical analysis, cases with weighted scores of more than 3 were defined as high expression, otherwise they were defined as low expression. Staining of LMP1 appeared on the cell membrane or/and in the cytoplasm. The expression of LMP1 was scored as positive (≥10 %) and negative (<10 %) based on the percentage of stained cells [23]. In order to detect the expressions of LMP1 and phosphorylated MSK1 at Thr581 in CNE1G and CNE1GL cells, they were immunocytochemically stained using the same method as for the clinical specimens.

### Cell proliferation assay

CNE1G and CNE1GL cells were seeded in 96-well plates (2 × 10^3^ cells per well) and incubated with different concentrations of H89 (Cell Signaling Technology, Beverly, MA). The si-mock or si-MSK1 stable CNE1 cells were transfected with pcDNA3.0 or pcDNA3.0-LMP1 plasmid and then seeded in 96-well plates with 100 μl cell suspension in each well (1 × 10^3^ cells per well). After culturing for various periods of time, 10 μl of CCK-8 solution (Dojindo Laboratories, Kumamoto, Japan) was added to each well, and cells were then incubated for 4 hour at 37 °C. Absorbance was measured at 450 nm using Synergy2 Multi-Mode Microplate Reader (BioTek, Winooski, VT, USA). The assay was conducted in five replicate wells for each sample and three parallel experiments were performed.

### Cell cycle analysis

CNE1 cells were seeded in 6-well plates and then transiently transfected with pcDNA3.0 or pcDNA3.0-LMP1 plasmid. H89 was added to the culture medium with different concentrations every 12 h after transfection. The si-mock or si-MSK1 stable CNE1 cells were transfected with pcDNA3.0 or pcDNA3.0-LMP1 plasmid and cultured for 48 h. The cells were harvested with trypsin, fixed with 70 % ice-cold ethanol, and stained with propidium iodide (PI). Then cell cycle distributions were analyzed by flow cytometry and the percentage of cells in G_1_/G_0_, S, or G_2_/M phase was calculated using ModFit LT software. Data represent the mean value derived from triplicate experiments.

### Colony forming assay

CNE1G and CNE1GL cells were seeded in 6-well plates (400 cells per well) and incubated with different concentrations of H89. After culturing for 2 weeks, colonies were fixed with methanol and stained with 0.5 % crystal violet. The number of colonies containing 50 cells or more was counted under an inverted microscope and the mean value from three replicate wells was calculated. Data are representative of at least three independent experiments.

### Anchorage-independent cell transformation assay

LMP1-induced cell transformation was investigated in si-mock or si-MSK1 stable CNE1 cells. In brief, the cells were transfected with pcDNA3.0 or pcDNA3.0-LMP1 plasmid and then seeded in 1 ml of 0.3 % basal medium Eagle’s agar containing 10 % FBS (800 cells per well). After culturing for 3 weeks, the number of colonies containing 50 cells or more was counted under an inverted microscope and the mean value from three replicate wells was calculated. Data are representative of at least three independent experiments.

### Reporter gene assay

*Fra-1* or *c-Jun* promoter activity and AP-1 activity were determined by the luciferase reporter gene assay. Cells were transiently cotransfected with various firefly luciferase reporter gene and pRL-TK vector (Promega, China). The pRL-TK vector expressing *Renilla* luciferase was cotransfected for normalizing the transfection efficiency. At 36 hours after transfection, cells were lysed with passive lysis buffer (Promega) for 20 min with gently shaking. The firefly luciferase and *Renilla* luciferase activities were measured with cell lysates using the Dual-Luciferase assay system (Promega) in FB12 Luminometer (Berthold detection system). The results were expressed as firefly luciferase activity normalized against *Renilla* luciferase activity. Data were derived from the mean value of triplicate samples and recorded as relative luciferase activity (fold or %). All experiments were done at least in triplicate.

### Protein extraction and western blot analysis

Cells were washed twice with cold PBS, and total protein was extracted with RIPA lysis buffer (Beyotime Ins. Bio, China) with protease inhibitor PMSF (Sangon, China). The isolation of histone was performed as described previously [18]. Protein concentration was determined by the bicinchoninic acid (BCA) assay (Pierce, Rockford, IL, USA). Samples containing equal amount of protein were resolved by SDS-PAGE and transferred to PVDF membranes. The membranes were probed with primary antibodies against phosphorylated or total MSK1(1:500), c-Jun (1:500), His-tag (1:500), histone H2A (1:1000) (all from Cell Signaling Technology, Beverly, MA), Fra-1(1:500, Santa Cruz Biotechnology, CA) and EBV LMP1 (CS1-4) (1:1000, DAKO, Glostrup), then incubated with infrared-dye-conjugated secondary antibodies (1:10000, Rockland Immunochemicals, Gilbertsville, PA) for 1 h at room temperature. Protein bands were visualized by Odyssey Infrared Imaging System (LI-COR Biotechnology, Lincoln, NE, USA) and quantified using the Quantity One software (Bio-Rad, USA).

### Statistical analysis

The SPSS version 16.0 software package and GraphPad Prism were used for the statistical analysis and data plotting. The Chi-square test was applied to examine the difference of phosphorylated MSK1 between NPC tissues and normal nasopharynx tissues as well as the relationship between phosphorylated MSK1 and LMP1 expression. Quantitative values were expressed as means ± SD. Student *t*-test was used to compare the mean value of each group. *P* < 0.05 was considered statistically significant.

## Results

### The level of MSK1 phosphorylation at Thr581 and its correlation with LMP1 in NPC tissues

**Fig. 1:**
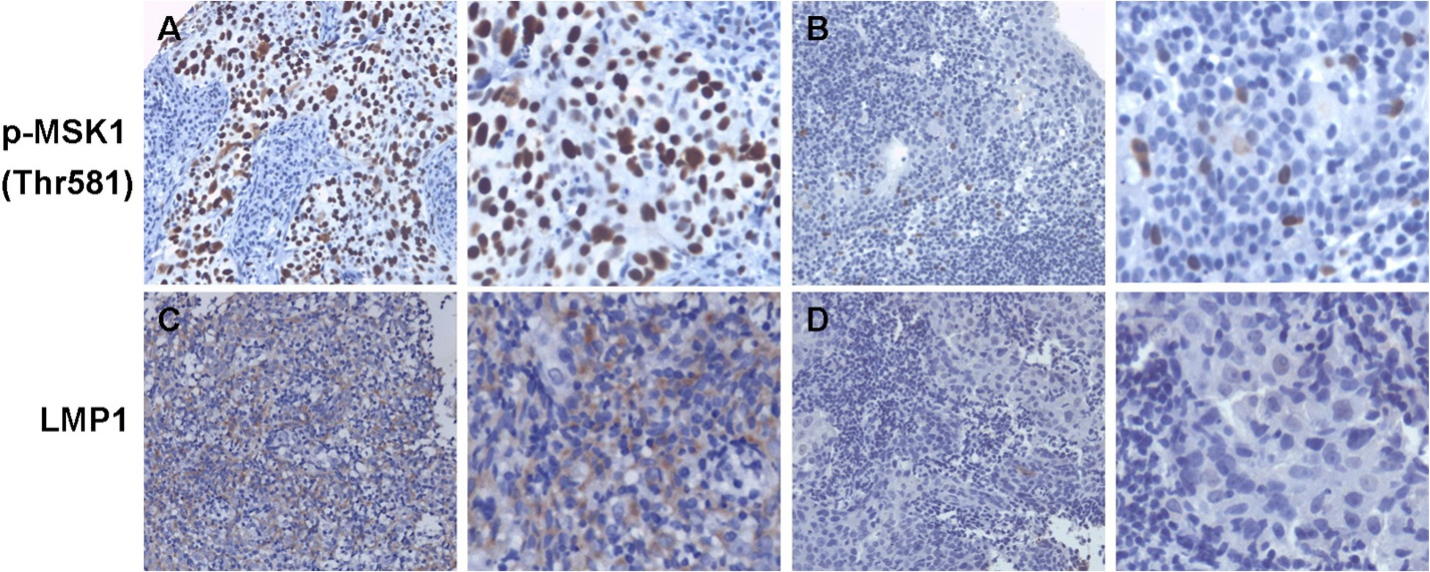
Expressions of phosphorylated MSK1 (Thr581) and LMP1 in nasopharyngeal carcinoma (NPC) and normal nasopharynx tissues by immunohistochemistry. (**a**) High expression of phosphorylated MSK1 (Thr581) in poorly differentiated NPC tissues. (**b**) Low expression of phosphorylated MSK1 (Thr581) in normal nasopharynx tissues. (**c**) Positive expression of LMP1 in poorly differentiated NPC tissues. (**d**) Negative expression of LMP1 in poorly differentiated NPC tissues. Images were captured at × 200 magnification (left) and × 400 magnification (right)

In order to assess the activation of MSK1 in the tumorigenesis of NPC, we analyzed the level of MSK1 phosphorylation at Thr581 in 53 archived paraffin-embedded NPC specimens and 36 adjacent/normal nasopharynx specimens using immunohistochemical staining. Phosphorylated MSK1 was expressed in cell nucleus (Fig. 1a, b). As shown in Table 1, the level of MSK1 phosphorylation at Thr581 was significantly higher in the poorly differentiated NPC tissues than that in normal nasopharynx tissues (*χ*^2^ = 15.137, *P* < 0.001). This revealed that the elevated phosphorylation of MSK1 at Thr581 might be involved in the malignant transformation of NPC cells.

**Table 1:** The level of phosphorylated MSK1 (Thr581) in nasopharyngeal carcinoma (NPC) and normal nasopharynx tissues

Sample	n	p-MSK1		*P*-value
		High	Low	
poorly differentiated NPC	53	29	24	<0.001
normal nasopharynx	36	5	21	

We further determined the relationship between phosphorylated MSK1 at Thr581 and LMP1 expression in 53 cases of NPC specimens. The LMP1 expression was located on cell membrane and cytoplasm (Fig. 1c, d). In NPC, 30 out of 53 (56.6 %) cases showed LMP1 expression. As shown in Table 2, there was a positive correlation between LMP1 expression and increased phosphorylation of MSK1 at Thr581 in NPC tissues (*r* = 0.393, *P* =0.002).

**Table 2:** Correlation between LMP1 expression and phosphorylated MSK1 (Thr581) in nasopharyngeal carcinoma (NPC) tissues

Groups		LMP1		r	*P*-value
		+	-		
p-MSK1	high	22	7	0.393	0.002
	low	8	16		

### LMP1 induces MSK1 activation via ERK-MAPK pathway in CNE1 cells

**Fig. 2:**
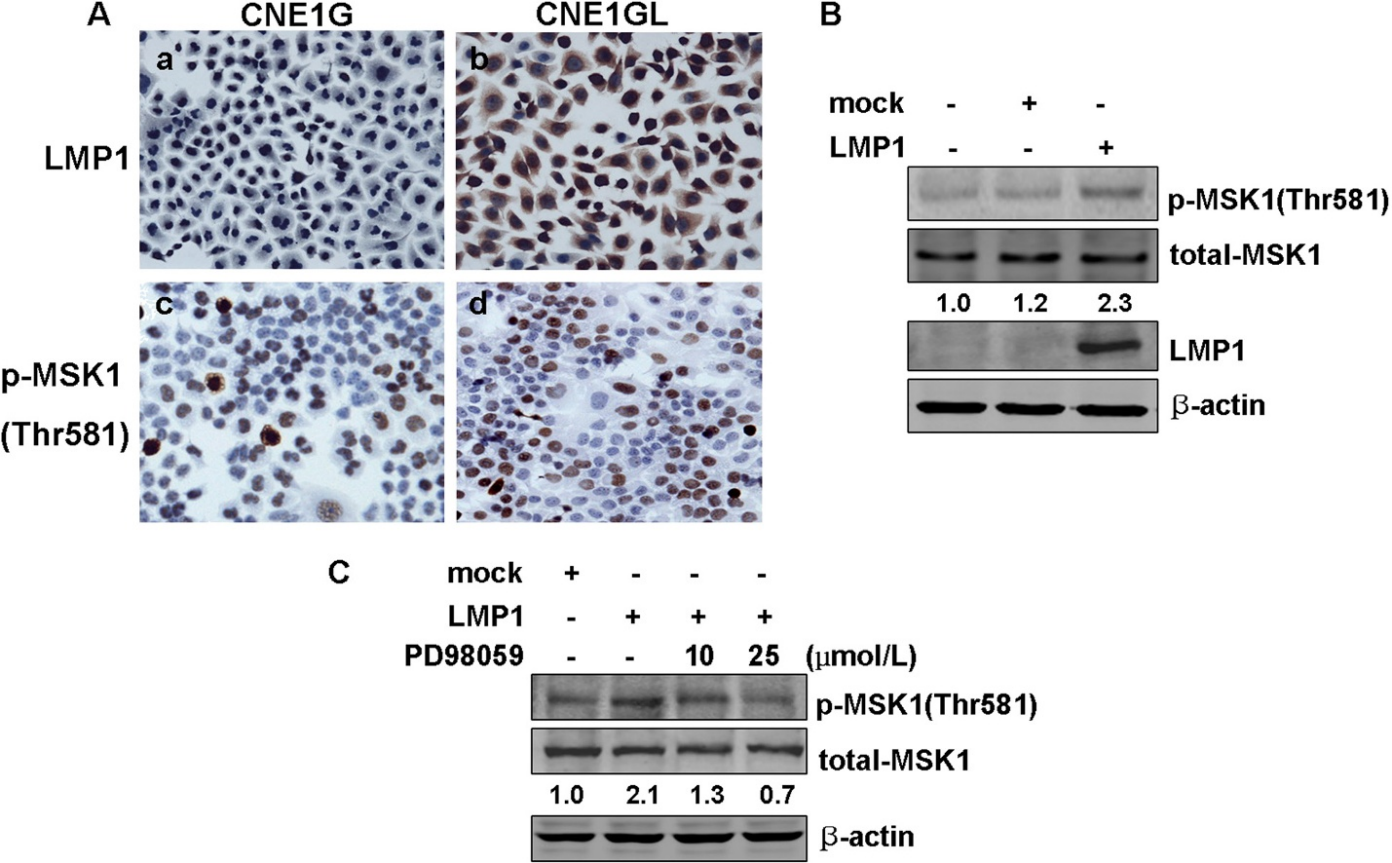
LMP1 induces phosphorylation of MSK1 at Thr581 in CNE1 cells. (**a**) CNE1G and CNE1GL cells were starved for 36 h by incubating in serum-deprived 1640 and subjected to immunocytochemical staining. No expression and positive staining of LMP1 (a, b) and different expression of phosphorylated MSK1 at Thr581 (c, d) in CNE1G and CNE1GL cells (magnification, ×200). (**b**) CNE1 cells were transfected with pcDNA3.0 or pcDNA3.0-LMP1. After 24 h of transfection, cells were starved for another 24 h by incubating in serum-deprived 1640 and then harvested. The expressions of LMP1 and phosphorylated MSK1 at Thr581 were detected by Western blotting. (**c**) CNE1 cells were transfected with pcDNA3.0 or pcDNA3.0-LMP1 vector. PD98059 was added to the culture medium at the concentration indicated every 12 h after transfection. The expression of phosphorylated MSK1 was detected by Western blotting. β-actin was used as loading control. Corresponding signaling intensities of phosphorylated MSK1 were densitometrically determined and normalized to total MSK1 in each lane and is given below in each data

To investigate whether LMP1 directly induced activation of MSK1 in NPC cells, we examined the relative level of phosphorylated MSK1 at Thr581 between CNE1G and CNE1GL cells by immunocytochemical staining. CNE1GL cells stably expressed LMP1 showed substantially high level of MSK1 phosphorylation compared with control CNE1G cells in the serum-starved condition (Fig. 2a), which was consistent with previous findings on MSK1 kinase activity [18]. In addition, CNE1 cells were transiently transfected with pcDNA3.0-LMP1, and the expression of phosphorylated MSK1 at Thr581 was examined by western blotting analysis. As shown in Fig. 2b, EBV-LMP1 could induce a dramatic increase of MSK1 phosphorylation at Thr581 in CNE1 cells. To further explore the signal mechanism mediated the activation of MSK1, PD98059, a specific ERK1/2 inhibitor, was used to treat the LMP1-transfected CNE1 cells. Our results showed that PD98059 obviously suppressed LMP1-induced phosphorylation of MSK1 at Thr581 in a dose-dependent manner (Fig. 2c).

### H89 inhibits LMP1-promoted CNE1 cell proliferation by delaying G_1_/S transition

**Fig. 3:**
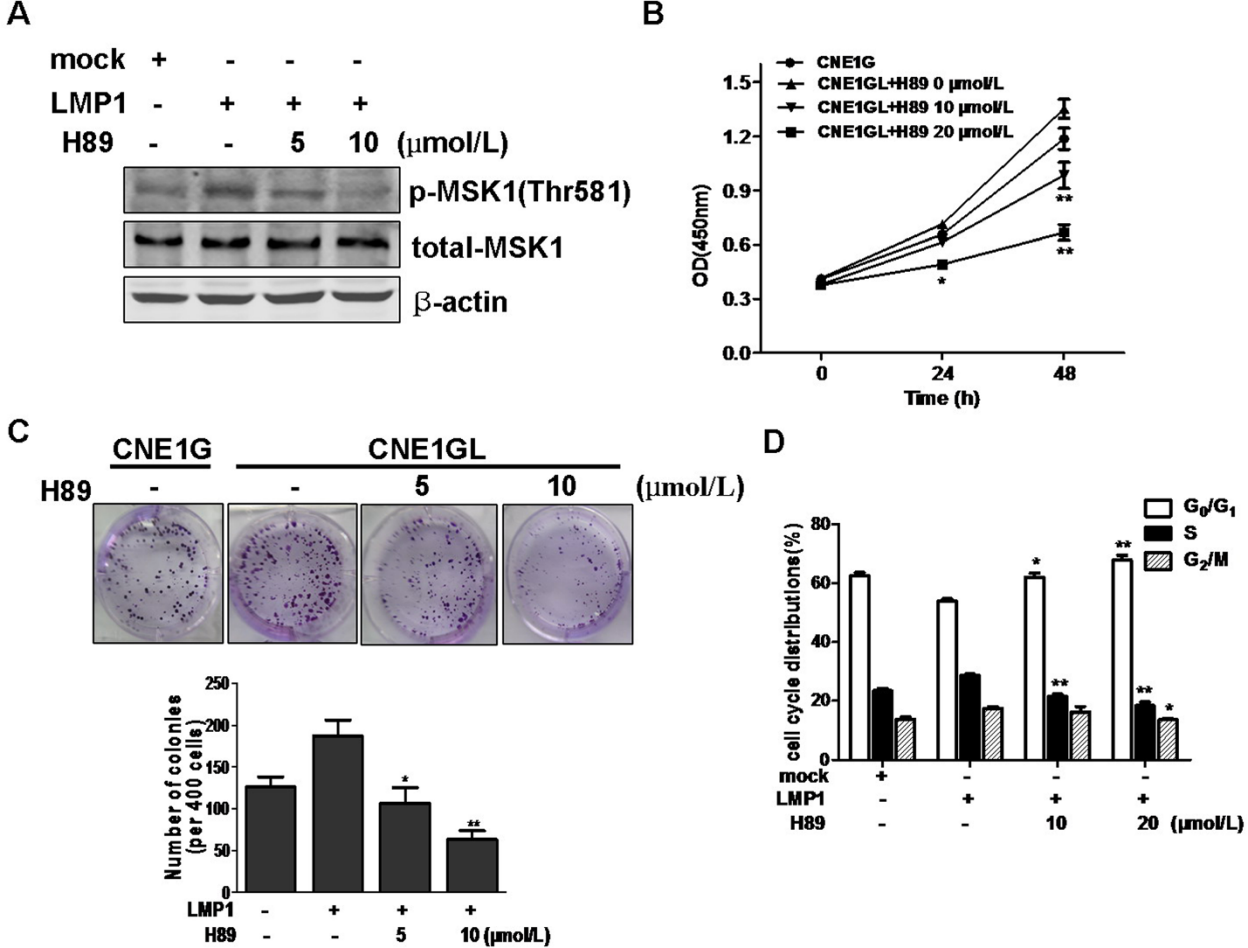
H89 inhibited LMP1-promoted CNE1 cell proliferation. (**a**) CNE1 cells were transfected with pcDNA3.0 or pcDNA3.0-LMP1. H89 was added to the culture medium at the concentration indicated every 12 h after transfection. The expression of phosphorylated MSK1 at Thr581 was detected by Western blotting. β-actin was used as loading control. (**b**) CNE1G and CNE1GL cells were seeded in 96-well plates and incubated with different concentrations of H89 for 24 h or 48 h. Cell proliferation was estimated using CCK-8 assay. Data are presented as mean ± SD for three independent experiments. *P < 0.05; **P < 0.001, compared with CNE1GL cells untreated with H89. (**c**) CNE1GL cells were incubated with different concentrations of H89 for 2 weeks, and then the ability of colony formation was evaluated. *P < 0.01; **P < 0.005, compared with CNE1GL cells untreated with H89. (**d**) The effect of H89 on LMP1-induced cell cycle distributions was analyzed by flow cytometric assay. Data are expressed as the percentage of cells in G_0_/G_1_, S, or G_2_/M phase. *P < 0.05; **P < 0.005, compared with LMP1-transfected CNE1 cells untreated with H89

To determine whether MSK1 activity is crucial for LMP1-promoted cell proliferation in NPC cells, H89, a potent inhibitor of MSK1, was used to treat the LMP1-transfected CNE1 cells. As expected, H89 markedly inhibited the LMP1-induced phosphorylation of MSK1 at Thr581 in a dose-dependent manner, but the total MSK1 protein level did not change (Fig. 3a). We assessed the effect of H89 on LMP1-promoted cell proliferation with different concentrations of H89 over varying periods. The results showed that H89 effectively suppressed LMP1-promoted cell proliferation in a time- and dose-dependent manner (Fig. 3b). In addition, H89 was found to dramatically block the colony formation promoted by LMP1 (Fig. 3c). The effect of H89 on cell cycle distributions was analyzed by flow cytometry. As shown in Fig. 3d, CNE1 cells expressing LMP1 showed a decrease of cells in G_0_/G_1_ phase_,_ and an increase of cells in the S and G_2_/M phases compared with mock cells, which indicated that LMP1 can accelerate cell cycle progression to promote the proliferation of CNE1 cells. However, the treatment of H89 caused a great accumulation of cells in the G_0_/G_1_ phase and a decrease in the S phase in a dose-dependent manner. These results suggested that H89 suppressed LMP1-promoted cell proliferation because of an impaired G_1_/S cell cycle transition.

### Knockdown of MSK1 suppresses LMP1-promoted CNE1 cell proliferation and anchorage-independent growth

**Fig. 4:**
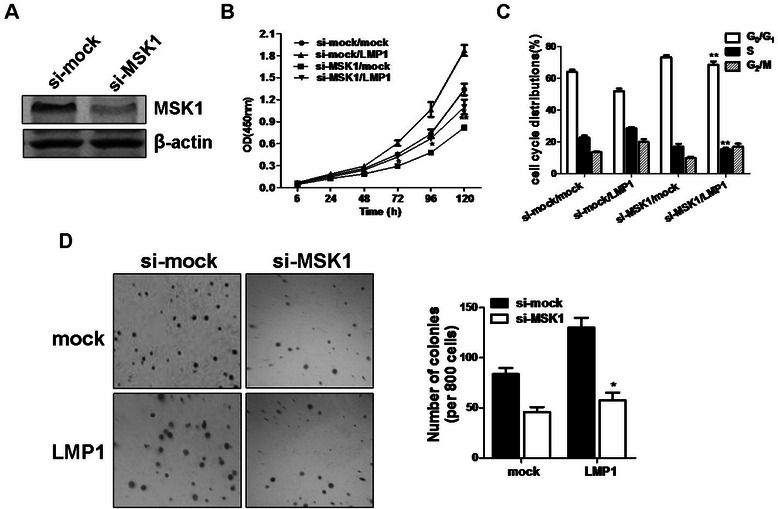
Knockdown of MSK1 suppressed LMP1-promoted CNE1 cell proliferation and anchorage-independent growth. (**a**) The expression of MSK1 was analyzed in the CNE1 cells stably transfected with si-mock or si-MSK1 by Western blotting. si-MSK1 efficiently suppressed the endogenous MSK1 protein level in CNE1 cells. (**b**) CNE1 cells stably expressing si-mock or si-MSK1 were transfected with pcDNA3.0 or pcDNA3.0-LMP1, and cell proliferation was estimated using CCK-8 assay. Data are presented as mean ± SD for three independent experiments. *P < 0.05; **P < 0.005, compared with si-mock/LMP1 cells. (**c**) The effect of MSK1 knockdown on LMP1-induced cell cycle distributions was analyzed by flow cytometric assay. Data are expressed as the percentage of cells in G_0_/G_1_, S, or G_2_/M phase. *P < 0.05; **P < 0.005, compared with si-mock/LMP1 cells. (**d**) CNE1 cells stably expressing si-mock or si-MSK1 were transfected with pcDNA3.0 or pcDNA3.0-LMP1, and soft agar assay was performed to detect the anchorage-independent growth capability. *P < 0.005, compared with si-mock/LMP1 cells

To ensure that the inhibition of cell proliferation was not a consequence of some other effect of H89, we designed siRNA against MSK1 (si-MSK1) and a scrambled control siRNA (si-mock) for transfection into CNE1 cells. Immunoblot analysis revealed that CNE1 cells stably transfected with si-MSK1 showed a suppression of endogenous MSK1 expression of up to 80 % compared with si-mock control cells (Fig. 4a). The si-mock or si-MSK1 stable CNE1 cells were transfected with pcDNA3.0 or pcDNA3.0-LMP1, and then cell proliferation and cell cycle profile were analyzed. Consistent to the effect of treatment with H89, the knockdown of MSK1 (si-MSK1/LMP1) markedly suppressed LMP1-promoted cell proliferation, and caused cell cycle arrest at the G_0_/G_1_ phase compared with the si-mock control cells (si-mock/LMP1) (Fig. 4b and 4c). Furthermore, the effect of MSK1 knockdown on anchorage-independent cell transformation was evaluated by soft agar assay. As shown in Fig. 4d, the knockdown of MSK1 strongly blocked the formation of transformed colonies induced by LMP1 compared with si-mock control cells. The inhibition was obvious not only in colony number, but also in colony size. These data indicated that MSK1 activity was required for LMP1-promoted malignant phenotype of CNE1 cells.

### MSK1 regulates LMP1-induced expressions of *Fra-1* and *c-Jun* in CNE1 cells

**Fig. 5:**
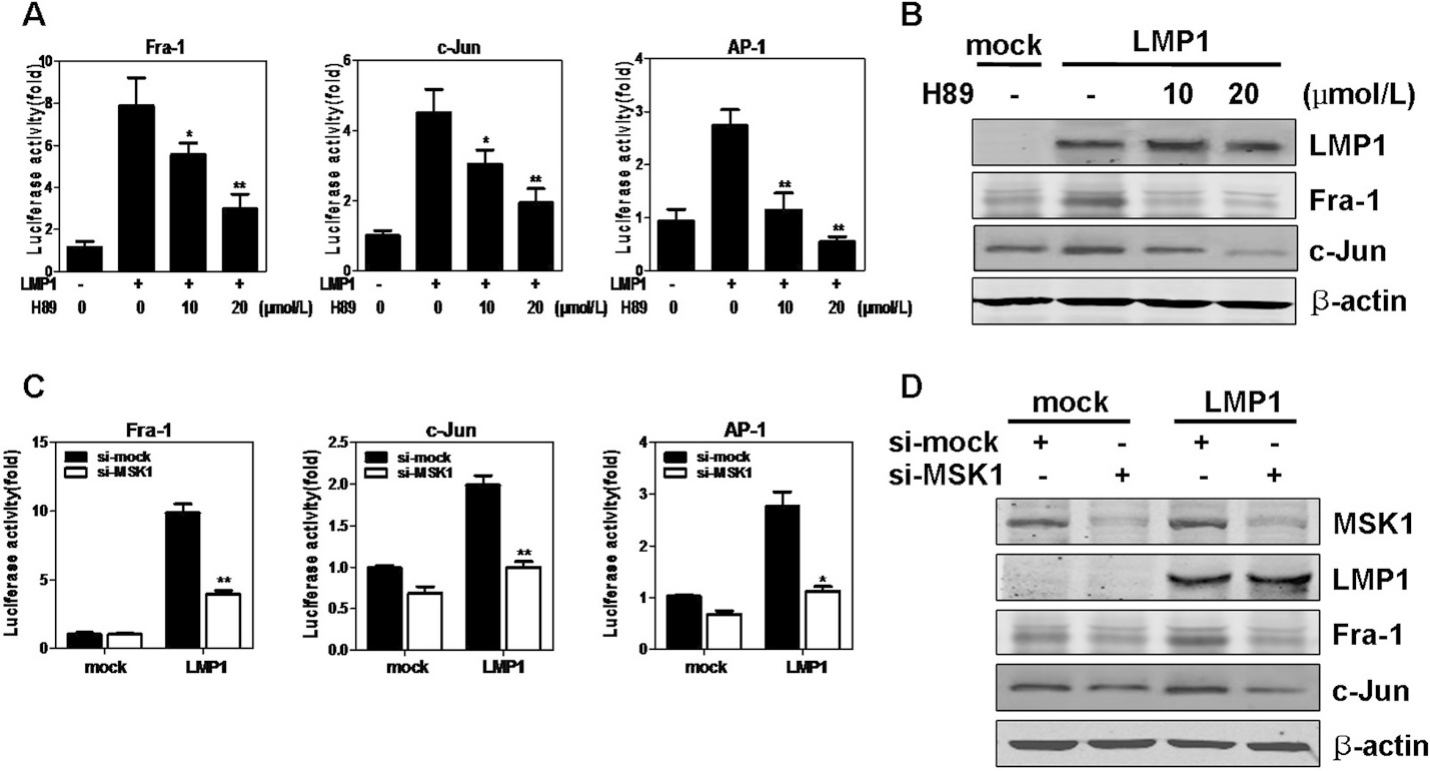
MSK1 regulates LMP1-induced transcriptional activation of *Fra-1* and *c-Jun* in CNE1 cells. (**a**) CNE1 cells were transfected with pcDNA3.0 or pcDNA3.0-LMP1. H89 was added to the culture medium at the concentration indicated every 12 h after transfection. The firefly luciferase activity was measured and normalized against *Renilla* luciferase activity respectively. Each bar represents the mean ± SD of three independent experiments. *P < 0.05; **P < 0.005, compared with LMP1-transfected CNE1 cells untreated with H89. (**b**) The effect of H89 on Fra-1 or c-Jun protein levels induced by LMP1 was examined by Western blotting. (**c**) CNE1 cells stably expressing si-mock or si-MSK1 were transfected with pcDNA3.0 or pcDNA3.0-LMP1. At 36 hours after transfection, the firefly luciferase activity was measured and normalized against *Renilla* luciferase activity respectively. *P < 0.01; **P < 0.005, compared with si-mock/LMP1 cells. (**d**) The effect of MSK1 knockdown on Fra-1 or c-Jun protein levels induced by LMP1 was examined by Western blotting. β-actin was used as loading control

MSK1 has been showed to play an important role in transcriptional and epigenetic regulation of genes in response to extracellular stimuli. We further explored whether the heightened activity of MSK1 is responsible for LMP1-induced gene activation involved in cell proliferation and transformation. LMP1 was found to upregulate activator protein (AP-1) transcriptional activity [24], but its mechanisms involved are still not completely clear. Examining the expressions of AP-1 proteins, we found that LMP1 can increase the expressions of endogenous Fra-1 and c-Jun proteins. However, the treatment of H89 dramatically suppressed the promoter activity of *Fra-1* or *c-Jun* induced by LMP1 in a dose-dependent manner (Fig. 5a), resulting in their decreasing protein levels (Fig. 5b). Next, we examined the effect of MSK1 knockdown on the induction of *Fra-1* or *c-Jun* gene*.* As shown in Fig. 5c and 5d, LMP1-promoted induction of *Fra-1* and *c-Jun* was effectively abrogated in si-MSK1 stably transfected cells compared with si-mock control cells. Furthermore, AP-1 activation induced by LMP1 was significantly blocked when MSK1 activity or expression was reduced in CNE1 cells, consistent to that observed for *Fra-1* or *c-Jun* activity. Taken together, these results showed that the increased MSK1 activity in LMP1-transformed CNE1 cells is critically important for induction of *Fra-1*, *c-Jun* promoter and AP-1 activation.

### Phosphorylation of histone H3 at Ser10 is involved in LMP1 induction of *Fra-1* and *c-Jun* in CNE1 cells

**Fig. 6:**
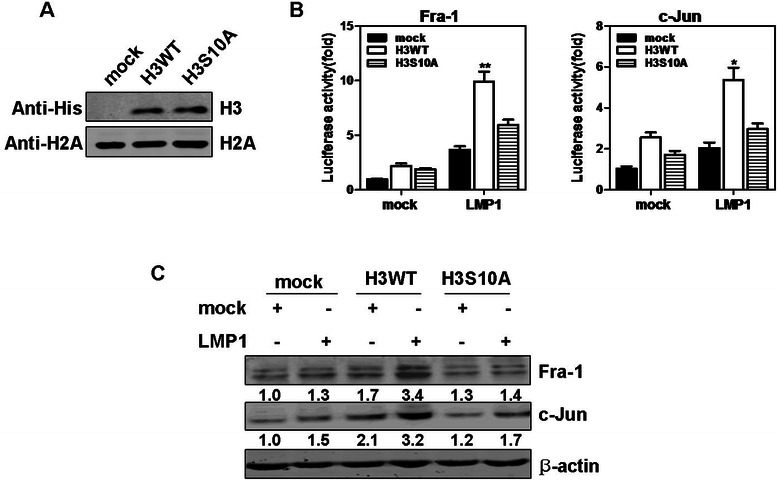
Phosphorylation of histone H3 at Ser10 is involved in LMP1 induction of *Fra-1* and *c-Jun* in CNE1 cells. (**a**) The expressions of H3 WT and H3 S10A mutant in stably transfected CNE1 cells were detected with an anti-His antibody against His-histone H3 by Western blotting. Histone H2A was used as loading control. (**b**) CNE1 cells stably expressing mock, H3 WT or mutant H3 S10A were transfected with pcDNA3.0 or pcDNA3.0-LMP1. At 36 hours after transfection, the firefly luciferase activity was measured and normalized against *Renilla* luciferase activity respectively. *P < 0.01; **P < 0.005, compared with mock/LMP1 cells. (**c**) The effect of overexpressing histone H3 WT or H3 S10A mutant on Fra-1 or c-Jun protein levels induced by LMP1 was examined by Western blotting. β-actin was used as loading control. Corresponding signaling intensities of Fra-1 or c-Jun were densitometrically determined and normalized to β-actin in each lane and is given below in each data

It was proposed that increased phosphorylation of histone H3 at Ser10 by activation of Ras-MAPK pathway and MSK1 may contribute to the aberrant gene expression observed in oncogene-transformed cells [25]. Our previous studies showed that MSK1 mediated LMP1-induced phosphorylation of histone H3 at Ser10 in CNE1 cells [18]. Thus, we further determined whether phosphorylation of histone H3 at Ser10 is involved in LMP1-promoted activation of *Fra-1* or *c-Jun*. The pcDNA6.0-H3 WT or mutant pcDNA6.0-H3 S10A plasmid was introduced into CNE1 cells (Fig. 6a), then the LMP1-promoted *Fra-1* or *c-Jun* promoter activity was assessed. As shown in Fig. 6b, LMP1-promoted transcriptional activity of *Fra-1* or *c-Jun* was significantly increased in CNE1 cells overexpressing H3 WT compared with mock control cells. In contrast, the histone H3 S10A mutant partly suppressed LMP1-promoted induction of *Fra-1* and *c-Jun* genes compared with H3 WT cells. We next detected the effect of histone H3 on the expression of Fra-1 or c-Jun proteins. Results showed that only H3 WT, but not H3 S10A, cells dramatically increased endogenous Fra-1 and c-Jun protein levels promoted by LMP1 compared with mock control cells (Fig. 6c). Taken together, these data suggested that histone H3 phosphorylation at Ser10 by MSK1 seems to be involved in LMP1-induced *Fra-1* and *c-Jun* gene activation in CNE1 cells.

## Discussion

MSK1 regulates transcription and cellular function at multiple levels and thereby exerts extensive effects in cell transformation, inflammation, neuronal plasticity, cardiac hypertrophy and other biological events [6]. MSK1 has been showed to be activated by either the ERK1/2 or p38 pathways depending on the specific stimuli. MSK1 activation is regulated by multiple phosphorylation sites. The phosphorylation at Thr581 located within the C-terminal kinase domain is essential for activation of MSK1. The mutation of Thr581 to an alanine residue prevented the activation of MSK1 in response to extracellular stimuli [26]. The elevated phosphoylation of MSK1 at Thr581 was observed in vulnerable neurons in Alzheimer's disease (AD) and the epidermis in lesional psoriatic skin, represented the activated ERK/p38 MAPK pathway and increased MSK1 activity [27, 28]. Various tumor promoters, such as EGF, TPA, or UVC and oncogenes have been showed to induce activation of MSK1 [29–31]. Furthermore, ~30 % of human cancers (*e.g.* colon, pancreatic, lung and breast cancers) have an overactive Ras-MAPK pathway and presumably elevated MSK1 activity even in the absence of external stimuli [14, 15]. In the present study, we found that the level of phosphorylated MSK1 at Thr581 was significantly high in poorly differentiated NPC tissues. It is indicated that the increased MSK1 activity might be an important event in the pathogenesis and progression of NPC.

LMP1, an important oncoprotein encoded by EBV, was found to be able to transform cell lines and induced multiple morphological and phenotypic alterations [4]. When expressed in epithelial cells, LMP1 exerted growth-promoting effects and modulated squamous epithelial differentiation [32, 33]. Functionally, LMP1 mimics a constitutively activated tumor necrosis factor receptor (TNFR), CD40, engaging a variety of signaling in a ligand-independent manner [34]. Activation of NF-κB, AP-1 and JAK3/STAT mediates various downstream pathological effects of LMP1 expression [5]. It has been demonstrated that LMP1 activates ERK-MAPK in epithelial cells via the canonical Raf-MEK-ERK-MAPK pathway in a Ras-independent manner [35]. In this study, we found that the elevated level of MSK1 phosphorylation at Thr581 in NPC tissues was closely related to LMP1 expression. Moreover, LMP1 induced phosphorylation of MSK1 at Thr581 under the serum-starved condition in CNE1 cells. But ERK1/2 inhibitor PD98059 obviously suppressed LMP1-induced phosphorylation of MSK1. The findings suggested that EBV-LMP1 could constitutively activate MSK1 via ERK1/2-MAPK signaling pathway in NPC.

It has been reported that MSK1 played a positive role in the control of cell proliferation of HaCaT keratinocytes [36] and EGF- or TPA-induced neoplastic cell transformation of JB6 Cl41 cells [29]. The inhibition of MSK1 activation significantly suppressed malignant phenotype of *Hras*-transformed cells [13]. Furthermore, MSK1/2 knockout mice developed significantly fewer skin tumors compared with wild-type mice in DMBA-initiated and TPA-promoted multistage skin carcinogenesis [37]. In the present study, we found that MSK1 directly regulated LMP1-promoted cell proliferation and transformation in CNE1 cells. When expressed in CNE1 cells, LMP1 can exert growth-promoting effects through accelerating cell cycle progression. However, the inhibition of MSK1 activity or expression dramatically suppressed LMP1-promoted cell proliferation by causing cell cycle arrest at G_1_-S phase transition. The malignant potential of LMP1-transformed CNE1 cells, represented by the anchorage-independent cell growth capability, was reduced by the knockdown of MSK1. These results indicated that MSK1 was involved in cell cycle regulation and its activity was required for LMP1-promoted cell proliferation and transformation in CNE1 cells.

The role of MSK1 in cancer progression is varied because of their many substrates and encompasses involvement in cell proliferation, transformation and inflammation [6, 38]. Increased MSK1 activity in *ras*-transformed mouse fibroblasts was required for maintaining the steady-state levels of Cox-2, Fra-1 and Jun and sustaining their ability to drive the cancer process [13]. MSK1 was involved in regulating the transcription of nuclear orphan receptor gene *Nur77*, *Nurr1* and *Nor1* [39], the up-regulation of which has been implicated in cell transformation [40]. The knockout of both MSK1 and MSK2 resulted in a 50 % reduction in *c-Fos* and *JunB* gene transcription in response to anisomycin or UVC radiation, but the transcription of *Egr-1* induced by anisomycin was not affected [7]. It has been reported that MSK1 activity was necessary for EGF- but not for TNFα-induced H3 Ser10 phosphorylation of the *c-Fos* promoter [41]. These studies strongly suggested that MSK1 might contribute to tumor promoter-induced cell transformation and tumorigenic progression through regulating the aberrant expression of specific genes. However, it remains to be resolved how MSK1 integrates the large variety of signals and accounts for the observed stimulus-, target gene- and cell-type-dependent specificity.

AP-1 is dimeric transcription factor mainly comprising members of Jun family (c-Jun、JunB, and JunD) and Fos family (c-Fos、FosB、Fra-1, and Fra-2). AP-1 dimers of different composition exert significant difference in DNA binding affinity and determine the target genes that it regulates [42]. AP-1 proteins are primarily considered to be oncogenic and have been reported to involve in cell transformation, proliferation, differentiation and apoptosis [42, 43]. The regulation of AP-1 proteins occurs at the level of transcription and/or post-transcriptional modifications. Fra-1, Fos-related antigen 1 encoding by *Fosl1*, dimerizes with Jun family proteins and thereby regulates various biological processes [44]. Fra-1 was strongly expressed in many kinds of epithelial tumors, and associated with poor prognosis of some squamous cell carcinomas [45, 46]. Fra-1 expression was also up-regulated in NPC cells compared with non-malignant nasopharyngeal epithelial cells [47]. Fra-1 was induced by EBV-LMP2A dependent on the activated ERK1/2 pathway and was essential for formation of AP-1 heterodimers mediating LMP2A-triggered MMP9 expression [48]. A potential link between LMP1 and Fra-1 comes from a previous study showing constitutive induction of Fra-1 in LMP1-transgenic mice [49]. c-Jun is a positive regulator of cell proliferation. The deficiency of c-Jun in fibroblasts leads to a marked proliferation defect due to the delay of cell cycle progression [50]. c-Jun played a pivotal role in Ras-induced cellular transformation and malignancy [51]*.* TNF-α-stimulated c-Jun expression preceded and was essential for subsequent Fra-1 induction in pulmonary epithelial cells [52]. Previous studies have been shown that LMP1 promoted the formation of c-Jun/JunB heterodimers leading to induction of AP-1 activation through JNK-mediated phosphorylation of c-Jun [53]. In this study, we found that LMP1 caused a significant increase in the transcriptional activity of *Fra-1* and *c-Jun* dependent on MSK1 activity. Chemical and genetic interference with MSK1 suppressed induction of *Fra-1* and *c-Jun* by LMP1, resulting in decreased AP-1 activity. These findings indicated that MSK1 activity was critical in maintaining LMP1-promoted expressions of *Fra-1* and *c-Jun*, which might alter AP-1 activation and its responsive genes.

Phosphorylation of histone H3 at Ser10 occurs in two difference phases of cell cycle with opposite functions. During mitosis, histone H3 Ser10 is globally phosphorylated by Aurora kinase, which facilitates compaction and transcriptional repression [54]. During interphase, extracellular stimuli also lead to phosphorylation of histone H3 in a minute fraction of histone H3, in correlation with rapid transcription induction of a subset of genes [55]. Accumulating evidences have demonstrated that phosphorylation of histone H3 at Ser10 in interphase is involved in different signaling pathways and effector kinases depending on specific stimulation and stress. Akt1, ERK2 and Ribosomal subunit protein S6 kinase 2 (RSK2), but not MSK1, were responsible for arsenite-induced phosphorylation of H3 at Ser10 [56], which was associated with induction of *Fos*, *Egr1* and *IL-8* [57]*.* It has been showed that PIM1 phosphorylated histone H3 at Ser10 on the nucleosome at the Myc-binding sites, contributing to transcriptional activation of *Fra-1* gene [58]. Our previous study has revealed that LMP1 can constitutively activate phosphorylation of histone H3 at Ser10 in interphase through the activation of Ras-MAPK pathway and MSK1 kinase in CNE1 cells [18]. In this study, we found that overexpression of histone H3 WT in CNE1 cells dramatically augmented LMP1 induction of *Fra-1* and *c-Jun* promoter activity compared with mock control cells, but the increase of activity was partly inhibited in the mutant H3 S10A cells. Constitutive activation of ERK-MSK1 pathway induced by LMP1 enhanced the level of phosphorylated histone H3 at Ser10 in H3 WT-overexpressing CNE1 cells. The elevated histone H3 phosphorylation may contribute to the decondensed chromatin struction and releasing blocks in elongation, which promotes the binding of transcription factors to the promoter region leading to the aberrant transcriptional activation of genes [55, 59]. The regulation of *Fra-1* or *c-Jun* gene is orchestrated by numerous transcription factors, which themselves must be activated. Moreover, other post-translational modifications of histone H3 such as methylation and acetylation might also be involved. Nonetheless, it has been suggested that phosphorylation of histone H3 at Ser10 mediated by MSK1 was closely linked with LMP1-induced transcriptional activation of *Fra-1* and *c-Jun* genes.

## Conclusions

In summary, these studies demonstrated a pivotal role of MSK1-mediated nucleosomal response in LMP1-promoted cell proliferation and transformation in NPC, which might be associated with its induction of aberrant gene expression. Considering that MSK1 is the active link between signaling cascades and the primary response of gene expression. It has been showed that MSK1/2 knock-out mice are viable and fertile and have no obvious health defects [7]. Therefore, MSK1 may consider as an ideal molecular target for cancer chemotherapy and gene therapy in NPC.

## Abbreviations

NPC: Nasopharyngeal carcinoma
EBV: Epstein-Barr virus
LMP1: Latent memberane protein 1
MSK1: Mitogen- and stress-activated kinase 1
AP-1: Activator protein-1
Fra-1: Fos-related antigen 1
siRNA: Small interfering RNA
